# Correction: Kwon, W.-A.; Lee, M.-K. Evolving Treatment Landscape of Frontline Therapy for Metastatic Urothelial Carcinoma: Current Insights and Future Perspectives. *Cancers* 2024, *16*, 4078

**DOI:** 10.3390/cancers17132097

**Published:** 2025-06-23

**Authors:** Whi-An Kwon, Min-Kyung Lee

**Affiliations:** 1 Department of Urology, Myongji Hospital, Hanyang University College of Medicine, Goyang-si 10475, Republic of Korea; 2 Department of Internal Medicine, Myongji Hospital, Hanyang University College of Medicine, Goyang-si 10475, Republic of Korea

## Error in Figure

In the original publication [1], there was a mistake in Figure 2b, where PD-1 and PD-L1 were switched. The corrected Figure 2 appears below. The authors apologize for any inconvenience caused and state that the scientific conclusions are unaffected. This correction was approved by the Academic Editor. The original publication has also been updated.

## Figures and Tables

**Figure 2:**
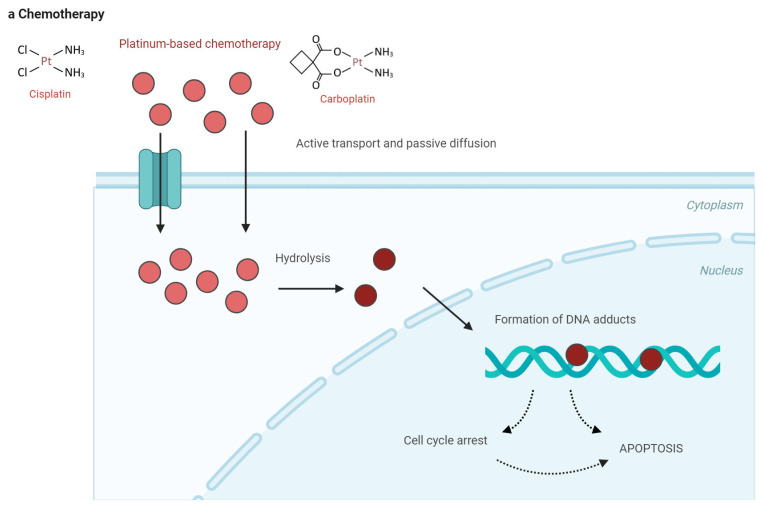

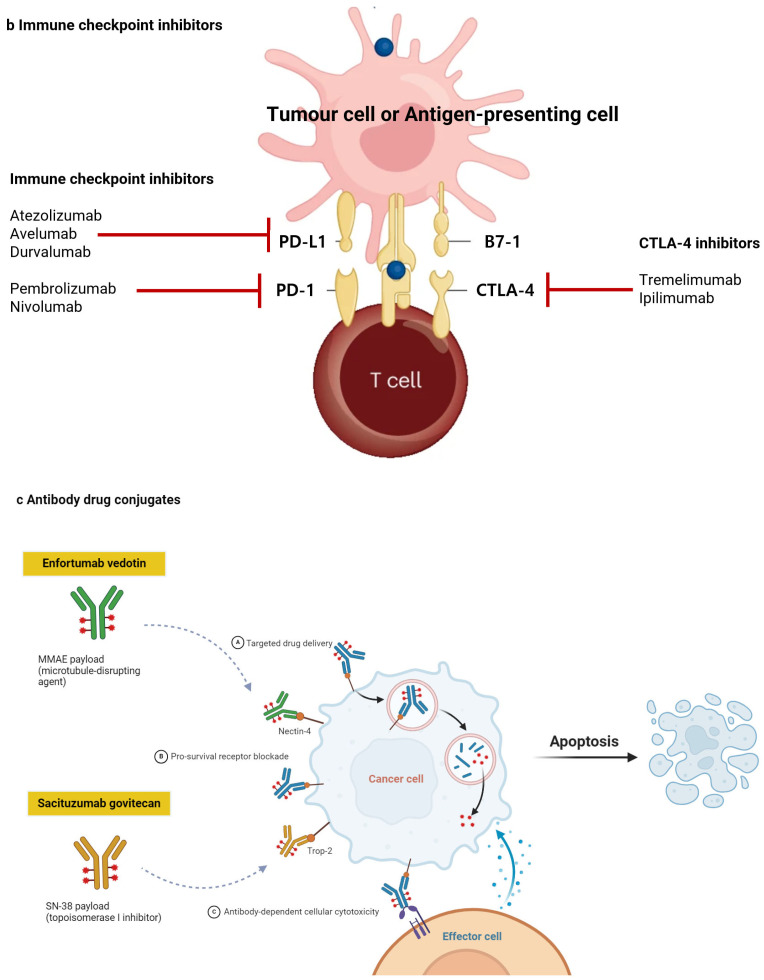
Drug classes and their mechanisms in the frontline treatment of metastatic urothelial carcinoma. (**a**) Platinum-based chemotherapy works by disrupting cell division. Platinum compounds bind to the DNA of cancer cells, creating cross-links that impede normal DNA replication and cell division. This DNA damage hampers the cancer cells’ growth and spread. (**b**) Immuno-therapy boosts the patient’s immune system to fight against cancer cells. It works by blocking immune checkpoint pathways, such as PD-1, PD-L1, and CTLA-4, which tumor cells use to evade immune detection. By inhibiting these pathways, immunotherapy enables the immune system to attack and eliminate cancer cells. (**c**) Antibody–drug conjugates (ADCs) consist of a cancer-specific antibody linked to a cytotoxic agent. Once the ADC attaches to the cancer cells, it is absorbed, and the toxic drug is released inside the cell, leading to cancer cell death. Enfortumab vedotin targets nectin-4, which is commonly overexpressed in urothelial cancer cells, while sacituzumab govitecan targets tumor cells through the anti-Trop-2 antibody. APC, antigen-presenting cell; CTLA-4, cyto-toxic T-lymphocyte associated protein 4; MMAE, monomethyl auristatin; PD-1, programmed cell death protein 1; PD-L1, programmed death-ligand 1; PD-L2, programmed death-ligand 2; SN-38, 7-ethyl-10-hydroxycamptothecin.
